# Combination therapy for pancreatic cancer: anti-PD-(L)1-based strategy

**DOI:** 10.1186/s13046-022-02273-w

**Published:** 2022-02-09

**Authors:** Lingyue Liu, Xing Huang, Fukang Shi, Jinyuan Song, Chengxiang Guo, Jiaqi Yang, Tingbo Liang, Xueli Bai

**Affiliations:** 1 Department of Hepatobiliary and Pancreatic Surgery, The First Affiliated Hospital, Zhejiang University School of Medicine, 79# Qingchun Road, Hangzhou, 310003 Zhejiang China; 2 Zhejiang Provincial Key Laboratory of Pancreatic Disease, The First Affiliated Hospital, Zhejiang University School of Medicine, Hangzhou, 310009 Zhejiang China; 3 Cancer Center, Zhejiang University, Hangzhou, 310058 Zhejiang China; 4 The Innovation Center for the Study of Pancreatic Diseases of Zhejiang Province, Hangzhou, 310009 Zhejiang China; 5 Zhejiang Clinical Research Center of Hepatobiliary and Pancreatic Diseases, Hangzhou, 310003 Zhejiang China

**Keywords:** Pancreatic cancer, Immune checkpoint inhibitor, PD-1, PD-L1, Combinational therapy, Systematic treatment, Post-translational modification, Precision therapy

## Abstract

Mortality associated with pancreatic cancer is among the highest of all malignancies, with a 5-year overall survival of 5–10%. Immunotherapy, represented by the blocking antibodies against programmed cell death protein 1 or its ligand 1 (anti-PD-(L)1), has achieved remarkable success in a number of malignancies. However, due to the immune-suppressive tumor microenvironment, the therapeutic efficacy of anti-PD-(L)1 in pancreatic cancer is far from expectation. To address such a fundamental issue, chemotherapy, radiotherapy, targeted therapy and even immunotherapy itself, have individually been attempted to combine with anti-PD-(L)1 in preclinical and clinical investigation. This review, with a particular focus on pancreatic cancer therapy, collects current anti-PD-(L)1-based combination strategy, highlights potential adverse effects of accumulative combination, and further points out future direction in optimization of combination, including targeting post-translational modification of PD-(L)1 and improving precision of treatment.

## Background

Mortality associated with pancreatic cancer (PC) is among the highest of all cancers, with a 5-year overall survival (OS) of only 5–10% and the highest incidence-to-mortality ratio of any solid tumor [1, 2]. It is anticipated that it will be the second greatest cause of cancer-related death by 2030 [3]. Because of the difficulties of early detection, the invasion of major blood vessels, aggressive biology characterized by early metastasis, and the wasting syndrome cachexia, the majority of patients with PC cannot undergo surgical resection, or relapse after surgery [4, 5]. Therefore, effective systemic treatments are urgently required. Although the mainstay of treatments for locally advanced or metastatic PC rely on systemic combination chemotherapy, specifically gemcitabine plus nab-paclitaxel or FOLFIRINOX (fluorouracil, leucovorin, irinotecan, and oxaliplatin) or modified FOLFIRINOX, survival has remained poor because of chemotherapeutic resistance, poor performance in patients, and the lack of biomarkers to match patients who tend to benefit from specific chemotherapeutic agents [6, 7].

Cancer immunotherapy has proved to be remarkably efficacious in a variety of malignancies. Traditionally, it aims to activate and increase the anti-tumor immune response in terms of both quantity and quality, which are generally categorized into two strategies [8]. The first category is passive immunotherapy, in which effector cells or molecules of the immune system directly attack tumor cells. Antibody-targeted therapy, antibody-drug conjugates, adoptive immune cell therapies, and engineered T cells such as chimeric antigen receptor (CAR) T cell and T cell receptor (TCR) T cell therapies are typical strategies [9, 10]. The second category is active immunotherapy, in which endogenous immune regulatory mechanisms are modulated to enhance anti-tumor immune activation. Using cancer vaccines and other agents are typical strategies, that enhance antigen uptake, processing, and presentation to T cells by antigen-presenting cells (APCs), enhancing the activation and expansion of naive T cells and effector T cells (Teffs) [11].

Over the last decade, monoclonal antibodies targeting programmed cell death protein 1 (PD-1, also known as PDCD1 and CD279) or programmed cell death ligand 1 (PD-L1, also known as CD274 and B7-H1) to restore tumor-induced immune deficiency have emerged as a promising treatment. Unlike previous immune enhancement strategies, anti-PD-(L)1 therapies block cancer immune evasion mechanisms, thereby promoting anti-tumor immune normalization [12]. Physiologically, the PD-L1-PD-1 axis attenuates the local T cell response and maintains immune homeostasis, signaling that cancers exploit to evade the anti-tumor immune response, and to facilitate tumor growth, progression, and metastasis [13, 14]. Therefore, blocking PD-(L)1 harnesses the endogenous anti-tumor response to fight cancer. This strategy has achieved a higher objective response rate (ORR) with considerably fewer immune-related adverse events (irAEs) in patients [8].

The PD-1 inhibitors currently approved by the US Food and Drug Administration (FDA) include pembrolizumab, nivolumab, and cemiplimab-rwlc. PD-L1 inhibitors approved by the FDA include atezolizumab, avelumab, and durvalumab [15]. These represent monoclonal antibodies that have demonstrated treatment efficacy against various solid and hematologic malignancies, including melanoma, non-small-cell lung cancer, head and neck squamous cell carcinoma, renal cell carcinoma, Hodgkin lymphoma, bladder cancer, and Merkel cell carcinoma [16, 17]. Nevertheless, the majority of PCs are resistant to anti-PD-(L)1 therapy due to their non-inflamed phenotype, namely immune-desert and immune-excluded phenotypes [18]. These tumors are characterized by a suppressive tumor microenvironment (TME) with low infiltration of Teffs combined with high infiltration of regulatory T cells (Tregs), tumor-associated macrophages (TAMs), and myeloid-derived suppressor cells (MDSCs). In addition, the dense fibrous stroma of a PC tumor impedes drug delivery and access of Teffs to its interior [11, 19]. A multicenter phase I trial in 2012 treated 207 cancer patients with an anti-PD-L1 antibody. Of 14 PC patients, no objective response was observed [20]. Another phase I trial which aimed to validate anti-PD-L1 (MPDL3280A) therapy included 1 PC patient who did not respond [21]. The greatest efficacy for the treatment of PC was reported from a phase I trial of pembrolizumab alone in which one patient achieved disease-free progression for 20 weeks [22]. Therefore, to overcome resistance to anti-PD-(L)1 monotherapy and integrate different anti-tumor therapeutic mechanisms, a variety of preclinical and clinical studies have been conducted to validate combining an anti-PD-(L)1 agent with chemotherapy, radiotherapy, targeted therapies, or other immunotherapies. The mechanisms and the details of preclinical trials, cases, and clinical trials **(**Table 1**)**, including ongoing trials **(**Table 2**)**, of various combinations are reviewed hereafter.

**Table 1:** Published clinical trials using anti-PD-(L)1 combination therapy

Publication year	Study phase	PC stage	Treatment line	Treatment regimens	Number of PC pts	Efficacy
Combination with chemotherapy						
2017	Ib/II	metastatic	1 / 2	pembrolizumab + gemcitabine, nab-paclitaxel	17	(Among the 11 chemotherapy naïve pts.) DCR 100%; mPFS 9.1 m, mOS 15.0 m
2020	I	locally advanced / metastatic	1 / 2	nivolumab + gemcitabine, nab-paclitaxel	50	(Among the 50 chemotherapy naïve pts.) ORR 18%, DCR 64%; mPFS 5.5 m, mOS 9.9 m
2020	/ (case)	metastatic	1	toripalimab + gemcitabine, nab-paclitaxel	1	PR for at least 8 cycles
Combination with radiotherapy						
2019	I/II	borderline resectable / locally advanced	2	durvalumab + stereotactic ablative radiotherapy	15	6 underwent R0 resection
2018	I	metastatic	≥ 2	pembrolizumab + hypofractionated radiotherapy	4	all PD
2017	Ib/II (randomized controlled study)	resectable / borderline resectable	1	pembrolizumab + radiation + capecitabine	22 (14 pts. in the triple therapy arm)	71% underwent surgery (compared to 50% in the arm only receiving capecitabine and radiation)
Combination with molecularly targeted therapy						
2019	II	metastatic	1	nivolumab + gemcitabine, nab-paclitaxel, cisplatin + paricalcitol	24	19 PR, 2 SD and 2 PD; mPFS 8.17 m, mOS 15.3 m
2018	I	locally advanced / metastatic		M7824 (a bifunctional fusion protein of anti-PD-L1 antibody fused to the extracellular domain of TGF-β receptor II)	5	1 durable PR (with dMMR), 1 prolonged SD
2019	Ib	metastatic		durvalumab + galunisertib (TGF-β receptor I kinase inhibitor)	32	1 PR, 7 SD; mPFS 1.9 m
2017	I	advanced	≥ 2	nivolumab + cabiralizumab (anti-CSF1R antibody)	31	3 PR (293, 275+, and 168+ days on study), 1 prolonged SD (182 d); 6-month DCR 13%, ORR 10%
2018	Ib/II	advanced		spartalizumab (anti-PD-1 antibody) + lacnotuzumab (anti-CSF1R antibody)	30	1 PR (on study for 346 d), 2 durable SD (on study for 328 d and 319 d)
2021	Ib	metastatic	1	nivolumab + APX005M (sotigalimab, CD40 agonist) + gemcitabine, nab-paclitaxel	12	8 PR, 3 SD; mPFS 10.8 m (0.1 mg/kg APX005M) / 12.4 m (0.3 mg/kg APX005M)
2020	I/II	metastatic	3 mean lines of prior treatment	pembrolizumab + NOX-A12 (CXCL12 inhibitor)	9	(among 11 colorectal cancer pts. and 9 PC pts.) 25% SD; mPFS 1.87 m, 6-month OS 42%, 12-month OS 22%
2019	IIb	metastatic	2 mean lines of prior treatment	pembrolizumab + BL-8040 (CXCR4 antagonist)	15	1 PR, 2 SD, DCR 21.4%; mOS 7 m
2020	IIa	metastatic	≥ 2	pembrolizumab + BL-8040 (CXCR4 antagonist)	37	(Among the 29 chemotherapy-resistant evaluable pts.) 1 PR, 9 SD, DCR 34.5%; mOS 3.3 m
			2	pembrolizumab + BL-8040 (CXCR4 antagonist) + liposomal irinotecan, fluorouracil, leucovorin	22	ORR 32%, DCR 77%; median duration of response 7.8 m
2018	I	?		pembrolizumab + defactinib (FAK inhibitor) + gemcitabine	8	1 PR; median time on treatment 127 d
2020	II (randomized controlled study)	locally advanced / metastatic	≥ 2	pembrolizumab + acalabrutinib (BTK inhibitor)	77 (40 pts. in the combination therapy arm)	ORR 7.9%, DCR 21.1%
2017	Ib	advanced		BGB-A317 (anti-PD-1 antibody) + BGB-290 (PARP 1 / 2 inhibitor)	? (38 pts. in all, including ovarian, breast, prostate, pancreatic cancers, etc.)	(among PC pts.) 1 PR, 2 SD (received the combination therapy for 189 d and 281 d)
2019	Ib	advanced		dostarlimab (PD-1 inhibitor) + niraparib (PARP inhibitor) + bevacizumab	?	1 SD in a PC pt
Combination with immunotherapy						
2020	/ (cases)	relapsed / refractory	complicated	nivolumab + ipilimumab	5	(among 3 evaluable pts.) 1 CR (with BRCA1), 1 PR (with RAD51C)
2019	II	metastatic	≥ 2	nivolumab + ipilimumab + radiation	25	ORR 13%, DCR 20%
2018	II (randomized controlled study)	metastatic	≥ 2	durvalumab + tremelimumab	64 (32 pts. in the combination therapy arm)	DCR 9.4%; mPFS 1.5 m, mOS 3.1 m
2018	II	metastatic	1	durvalumab + tremelimumab + gemcitabine, nab-paclitaxel	11	PR 73%, DCR 100%; mPFS 7.9 m, 6-month survival 80%
2018	I	advanced	≥ 2	pembrolizumab + epacadostat (IDO1 inhibitor)	1	PR (on treatment at 21 w)
2019	I	advanced		atezolizumab + navoximod (IDO1 inhibitor)	1	PR
2015	/	metastatic	2	antigen-primed MoDCs modified by PD-L1 blockade	10	5 pts. (didn’t respond to MoDC alone) achieved secondary stabilization (4 m to 8 m) by using MoDCs modified by PD-L1 blockade
2016	/	metastatic		nivolumab + antigen-primed MoDCs	7	2 PR
2019	Ib	advanced	2	pembrolizumab + pelareorep (oncolytic reovirus) + chemotherapy	11	(among 10 efficacy-evaluable pts.) 1 PR for 17.4 m, 2 SD lasting 9 m and 4 m

Abbreviations: BTK, Bruton’s tyrosine kinase; CSF1R, colony-stimulating factor 1 receptor; CXCL12, CXC-chemokine ligand 12; CXCR4, CXC-chemokine receptor 4; DCR, disease control rate; dMMR, mismatch repair-deficient; FAK, focal adhesion kinase; IDO1, indoleamine 2,3-dioxygenase 1; MoDC, monocyte-derived dendritic cell; ORR, overall response rate; OS, overall survival; PARP, poly ADP-ribose polymerase; PC, pancreatic cancer; PD, progressive disease; PD-(L)1, programmed cell death (ligand) 1; PFS, progression-free survival; PR, partial response; pts., patients; SD, stable disease; TGF-β, transforming growth factor-β

**Table 2:** Ongoing clinical trials using anti-PD-(L)1 combination therapies^a^

Anti-PD-(L)1 regimen	Combination regimens	PC stage	Treatment line	Primary outcomes	Study phase	Trial ID
nivolumab	FOLFIRINOX	resectable / borderline resectable	1	clinically relevant pancreatic fistula in the post-operative period, pCR	I/II	NCT03970252
	SBRT	locally advanced	2	incidence of TRAEs, incidence of laboratory abnormalities	I/II	NCT04098432
	irreversible electroporation + CpG (toll-like receptor 9 ligand)	metastatic	≥ 2	TRAEs	I	NCT04612530
	irreversible electroporation					
	SX-682 (CXCR1 / 2 inhibitor)	metastatic	≥ 2	MTD	I	NCT04477343
	cabiralizumab (CSF1R inhibitor) + gemcitabine	metastatic	2	PFS	II	NCT03697564
	APX005M (CD40 agonist) + GA	metastatic	1	number and percentage of subjects with AEs, SAEs, DLTs; OS	Ib/II	NCT03214250
	entinostat (class I histone deacetylases inhibitor)	advanced		ORR	II	NCT03250273
	TPST-1120 (peroxisome proliferator activated receptor alpha antagonist)	advanced		incidence of DLTs, TEAEs; MTD	I	NCT03829436
	autologous dendritic cell vaccine loaded with personalized peptides + gemcitabine, capecitabine	resectable		number of cases for which vaccine is produced, AEs	Ib	NCT04627246
	KRAS peptide vaccine + ipilimumab	resected		number of participants experiencing DRTs, fold change in interferon-producing mutant-KRAS-specific CD8 and CD4 T cells at 16 w	I	NCT04117087
	GVAX + SBRT + cyclophosphamide	borderline resectable	no more than 1 month / cycle (28 days) of systemic therapy	CD8 count in the TME	II	NCT03161379
	GVAX + cyclophosphamide	resectable	1	median IL-17A expression in vaccine-induced lymphoid aggregates found in surgically resected PC	I/II	NCT02451982
	GVAX + urelumab (CD137 agonist) + cyclophosphamide					
	GVAX + BMS-813160 (CCR2 / CCR5 antagonist) + SBRT	locally advanced	1	number of participants experiencing DRTs, percentage of participants who achieve an immune response (> 80% increase of infiltration of CD8 ^+^ CD137^+^ T cells into the tumor)	I/II	NCT03767582
	BMS-813160 + SBRT					
	ipilimumab + CRS-207 (live, attenuated *Listeria monocytogenes*-expressing mesothelin) + GVAX + cyclophosphamide	metastatic		ORR	II	NCT03190265
	ipilimumab + CRS-207					
	GRT-C903 (shared neoantigen cancer vaccine prime) + GRT-R904 (shared neoantigen cancer vaccine boost) + ipilimumab	advanced	2	incidence of AEs, SAEs, DLTs; RP2D of GRT-C903 and GRT-R904; ORR in phase 2	I/II	NCT03953235
pembrolizumab	INT230–6 (comprised of 3 agents: cisplatin, vinblastine sulfate, and a cell permeation enhancer)	advanced	no limit	rate and severity of ≥ grade 3 TEAEs	I/II	NCT03058289
	PEGPH20	metastatic	2	PFS	II	NCT03634332
	efineptakin alfa (NT-I7, long-acting human IL-7)	advanced	2	incidence, nature and severity of AEs, incidence and nature of DLTs, MTD and RP2D of NT-I7; ORR in phase IIa	Ib/IIa	NCT04332653
	GB1275 (modulator of CD11b)	metastatic		incidence of DLTs, AEs; maximum plasma concentration, trough plasma concentration, time of maximum observed plasma concentration, terminal phase elimination half-life, area under the plasma concentration-time curve, oral clearance of GB1275; ORR in phase II	I/II	NCT04060342
	ENB003 (endothelin B receptor antagonist)	metastatic	≥ 2	incidence of TEAEs, ORR	Ib/IIa	NCT04205227
	itacitinib (INCB039110, JAK1 inhibitor)	advanced		frequency, duration, and severity of AEs	Ib	NCT02646748
	INCB050465 (PI3K-delta inhibitor)					
	lenvatinib	advanced	≥ 2	ORR; percentage of participants who experience an AE or discontinue treatment due to an AE	II	NCT03797326
	Debio 1143 (a second mitochondrial-derived activator of caspases mimetic designed to promote apoptosis)	Stage III or IV	≥ 2	MTD, RP2D, extension part ORR	I	NCT03871959
	defactinib (focal adhesion kinase inhibitor)	advanced	≥ 2	AEs, MTD	I/IIa	NCT02758587
	defactinib + gemcitabine	advanced	2	RP2D (determined from MTD)	I	NCT02546531
	defactinib + chemotherapy	resectable	1	pCR rate	II	NCT03727880
	olaparib (PARP inhibitor) + GAX-CI (gemcitabine, nab-paclitaxel, capecitabine, cisplatin, and irinotecan)	metastatic	1	PFS after 6 m	II	NCT04753879
	ipilimumab + anetumab ravtansine (an anti-mesothelin antibody conjugated to the maytansinoid tubulin inhibitor DM4)	advanced	≥ 2	MTD	I	NCT03816358
	anetumab ravtansine + gemcitabine					
	GVAX + IMC-CS4 (LY3022855, anti-CSF1R antibody) + cyclophosphamide	borderline resectable		CD8 T cell density in the primary tumor, number of participants experiencing DRTs	I	NCT03153410
	epacadostat + GVAX + cyclophosphamide	metastatic	≥ 2	recommended dose of epacadostat; 6-month survival	II	NCT03006302
	epacadostat + CRS-207 (live, attenuated *Listeria monocytogenes*-expressing mesothelin)					
	GVAX + cyclophosphamide + SBRT	locally advanced	≥ 2	distant metastasis free survival	II	NCT02648282
	p53MVA vaccine	advanced	≥ 2 or refuse standard treatment	tolerability of the combination	I	NCT02432963
	mRNA-5671/V941	advanced		DLTs, AEs, discontinuations	I	NCT03948763
pembrolizumab (local delivery via trans-artery or intra-tumor injection)	ipilimumab (local delivery via trans-artery or intra-tumor injection)	advanced		OS, CR rate before or at 6 m	II/III	NCT03755739
spartalizumab	NIS793 (anti-TGF-β antibody)	advanced		incidence of DLTs, AEs, SAEs, dose reductions/interruptions	I	NCT02947165
	NIS793 + GA	metastatic	1	incidence of DLTs, incidence and severity of TEAEs and SAEs, dose interruptions / reductions, dose intensity; PFS	II	NCT04390763
	canakinumab (ACZ885, anti-IL-1β antibody) + GA	metastatic	1	recommended phase 2 / 3 dose	Ib	NCT04581343
dostarlimab	niraparib (PARP inhibitor)	metastatic	2 or 3	DCR at 12w	II	NCT04493060
sintilimab	mFOLFIRINOX	metastatic	1 or 2	OS	III	NCT03977272
toripalimab	mFOLFIRINOX	borderline resectable / locally advanced		PFS	III	NCT03983057
	anlotinib + nab-paclitaxel	locally advanced / metastatic	2	PFS	II	NCT04718701
camrelizumab	GA	metastatic	1	PFS	III	NCT04674956
	GA	metastatic		ORR, PFS	II	NCT04498689
	plerixafor (AMD3100, CXCR4 antagonist)	metastatic	≥ 2	ORR	II	NCT04177810
zimberelimab	AB680 (CD73 inhibitor) + GA	metastatic		number of participants with TEAEs	I	NCT04104672
unknown PD-1 antibody	radiotherapy	advanced		local control	II	NCT03374293
	GA + manganese	locally advanced / metastatic		number of subjects with TRAEs, DCR	I/II	NCT03989310
	GA					
	LYT-200 (galectin-9 inhibitor) + GA	metastatic		incidence of TEAEs and DLTs, PFS, ORR	I/II	NCT04666688
	apatinib (VEGFR-2 tyrosine kinase inhibitor) + radiation	advanced		PFS	/	NCT04365049
	mutant KRAS G12V-specific TCR transduced autologous T cells + cyclophosphamide, fludarabine	advanced		frequency and severity of TRAEs, ORR	I/II	NCT04146298
durvalumab	danvatirsen (antisense oligonucleotide targeting signal transducer and activator of transcription 3)	refractory / stage II / stage III / stage IV		incidence of AEs, SAEs; physiological parameters; incidence of TEAEs and deaths; PD-L1 expression; phosphorylated or total STAT3 expression levels; characterization of immune infiltrates; quantification and characterization of CD8 staining pattern; PD-L1 protein levels in the membrane of circulating tumor cells; physical examinations	II	NCT02983578
	olaparib (PARP inhibitor)	advanced		changes in genomic and immune biomarkers	II	NCT03851614
	cediranib (inhibitor of VEGFR tyrosine kinases)					
	tremelimumab + SBRT	advanced	≥ 2	number of AEs with grade 1–5	I/II	NCT02311361
	SBRT					
	CV301 (poxviral-based vaccine) + capecitabine	metastatic	2	RP2D of durvalumab, 8.5-month PFS rate, 4-month PFS rate	I/II	NCT03376659
atezolizumab	BDB001 (Toll-like receptor agonist) + radiotherapy	metastatic		DCR within 24 w	II	NCT03915678
	KY1044 (anti-ICOS antibody)	advanced		incidence and severity of AEs and SAEs; number of dose interruptions, reductions and dose intensity; ORR	I/II	NCT03829501
	personalized neoantigen tumor vaccines + mFOLFIRINOX	resectable	1	DRT	I	NCT04161755
	LOAd703 (oncolytic adenovirus encoding TMZ-CD40L and 4-1BBL) + GA	advanced		number of patients with DLTs	I/IIa	NCT02705196
avelumab	binimetinib (MEK inhibitor) + talazoparib (PARP inhibitor)	locally advanced / metastatic	2 or 3	DLT, confirmed objective response	Ib/II	NCT03637491
	binimetinib					
	ETBX-011 (vaccine inducing CEA-specific cytotoxic T-cell activity) + GI-4000 (vaccine expressing mutant Ras proteins) + haNK + ALT-803 (IL-15 superagonist) + bevacizumab + aldoxorubicin HCl, capecitabine, cyclophosphamide, fluorouracil, leucovorin, nab-paclitaxel, omega-3-acid ehtyl esters, oxaliplatin + SBRT	advanced	≥ 2	incidence of TEAEs and SAEs, ORR	Ib/II	NCT03387098^b^
	ETBX-011 + ETBX-021 (HER2) + ETBX-051 (Brachyury) + ETBX-061 (MUC1) + GI-4000 + GI-6207 (CEA yeast) + GI-6301 (Brachyury yeast) + haNK + ALT-803 + bevacizumab + aldoxorubicin HCl, capecitabine, cyclophosphamide, fluorouracil, leucovorin, nab-paclitaxel, oxaliplatin + SBRT	advanced	≥ 2	incidence of TEAEs and TESAEs, ORR	Ib/II	NCT03586869
LY3300054 (anti-PD-L1 antibody)	merestinib (multikinase inhibitor)	locally advanced / metastatic	1	number of participants with LY3300054 DLTs	I	NCT02791334
SHR-1701 (PD-L1/TGF-β bsAb)	GA	advanced	1	RP2D, ORR	Ib/II	NCT04624217
PD-L1/CTLA-4 bsAb	/	locally advanced / metastatic	2	ORR	I/II	NCT04324307
	GA		1			
	FOLFIRINOX		1			
PD-L1 targeting haNK	N-803 (IL-15 superagonist) + SBRT + cyclophosphamide, gemcitabine, nab-paclitaxel, aldoxorubicin HCl	locally advanced / metastatic	≥ 2	PFS	II	NCT04390399

^a^Abbreviations: AE, adverse effect; bsAb, bispecific antibody; CR, complete response; DCR, disease control rate; DLT, dose-limiting toxicity; DRT, drug-related toxicity; FOLFIRINOX, fluorouracil, leucovorin, irinotecan, and oxaliplatin; GA, gemcitabine, nab-paclitaxel; GVAX, granulocyte-macrophage colony-stimulating factor-secreting PC vaccine; haNK, high-affinity natural killer; mFOLFIRINOX, modified FOLFIRINOX; MTD, maximum tolerated dose; ORR, overall response rate; OS, overall survival; PARP, poly (ADP-ribose) polymerase; PC, pancreatic cancer; pCR, pathologic complete response; PD-(L)1, programmed cell death (ligand) 1; PEGPH20, PEGylated recombinant hyaluronidase PH20; PFS, progression-free survival; RP2D, recommended phase 2 dose; SAE, severe adverse effect; SBRT, stereotactic body radiation therapy; TEAE, treatment-emergent adverse event; TESAE, treatment-emergent severe adverse event; TME, tumor microenvironment; TRAE, treatment-related adverse event; VEGFR, vascular endothelial growth factor receptor

^b^This group also initiated 2 studies exploring combination therapies without aldoxorubicin HCl, or without aldoxorubicin HCl + omega-3-acid ethyl esters + SBRT (other regimens are the same; trial ID NCT03329248; NCT03136406)

## Combination with chemotherapy

In patients with locally advanced or metastatic PC, gemcitabine plus nab-paclitaxel, FOLFIRINOX, and modified FOLFIRINOX are considered first-line treatment regimens for those with good performance status [23, 24]. While nab-paclitaxel has been shown to enhance the release of granzyme B by effector cells and cause the desmoplastic stroma to increase intratumoral gemcitabine penetration, gemcitabine can increase antigen presentation by upregulating major histocompatibility complex (MHC) class I expression and promote dendritic cell maturation [25, 26]. Moreover, FOLFIRINOX has been shown to reduce human leukocyte antigen-A defects, increase CD8^+^ cell numbers, and decrease Treg and M2 macrophage density [27]. The expression of the immunosuppressive gene CXCL5 has been shown to be reduced after delivering FOLFIRINOX [28]. These findings demonstrate that in addition to a direct cytotoxic effect on cancer cells, PC chemotherapy may promote immunogenic cell death (ICD), which provides the foundation for combination therapy with an anti-PD-(L)1 agent. Significantly, PD-L1 expression may be influenced by the type of chemotherapy and the response to treatment, which in return will influence the efficacy of an anti-PD-(L)1 combination therapy [29–31].

Preclinical studies have provided supporting evidence for combining anti-PD-(L)1 therapy with chemotherapy for PC. In a transgenic mouse model of resectable PC, a neoadjuvant PD-1 antagonist (150 mg/mouse at day 0, 3, 6; clone RMP1–14) with gemcitabine (100 mg/kg at day 0, 3, 6) resulted in significantly improved survival compared with neoadjuvant gemcitabine alone. Following combination therapy, tumor infiltration by neoantigen-specific CD8^+^ T lymphocytes against the marker neoepitope LAMA4-G1254V and CD103^+^CD8^+^ T cells increased, confirming local T cell activation [32]. In an additional study using a syngeneic subcutaneous PC mouse model, the anti-tumor properties of gemcitabine (60 μg/g Q3D, 0–2 weeks) were enhanced by combining with delayed 2-week PD-L1 blockade (0.3 mg TIW, 2–4 weeks). Furthermore, gemcitabine (using the same dosing strategy) with simultaneous and subsequent 4-week PD-L1 blockade (0.3 mg TIW, 0–4 weeks) displayed a substantial synergistic anti-tumor effect and resulted in complete response (CR) [33]. Moreover, in a syngeneic orthotopic PC mouse model, the combination of PD-L1 blockade (100 μg BIW for 2 weeks, clone 10F.9G2) with urokinase plasminogen activator receptor-targeted iron oxide nanoparticles loaded with cisplatin (10 mg/kg drug equivalent) decreased tumor burden by ~ 65% [34]. In a syngeneic subcutaneous PC mouse model, the combination of the novel taxoid DHA-SBT-1214 (25 mg/kg QW for 6 weeks) and anti-PD-L1 antibody (200 μg BIW for 3 weeks, clone 10F.9G2) also resulted in enhanced CD8^+^ T cell infiltration that provided therapeutic effects [35].

When used clinically, the addition of pembrolizumab to chemotherapy has displayed slightly improved efficacy. In a single-center phase Ib/II study, 11 chemotherapy-naive metastatic PC patients were administered gemcitabine (1000 mg/m^2^ on day 1 and 8 every 3 weeks), nab-paclitaxel (125 mg/m^2^ on day 1 and 8 every 3 weeks), and pembrolizumab (2 mg/kg Q3W). Their median progression-free survival (mPFS) and median overall survival (mOS) were 9.1 and 15.0 months, respectively. The disease control rate (DCR) was 100%, within which 3 patients displayed a partial response (PR) for 8+, ~ 11, and 15 months. In that study, DNA copy number instability was considered prognostic for OS [36]. However, disappointing outcomes were observed in a phase I study combining nivolumab (3 mg/kg on day 1 and 15 every 4 weeks) with gemcitabine (1000 mg/m^2^ on day 1, 8, 15 every 4 weeks) and nab-paclitaxel (125 mg/m^2^ on day 1, 8, 15 every 4 weeks) in patients with locally advanced or metastatic PC. Of the 50 treated patients, mPFS and mOS were 5.5 and 9.9 months, respectively. The ORR was 18% (1 CR and 8 PR) while DCR was 64%. These study results of efficacy did not support further investigation [37]. Additionally, PD-L1 positive expression was detected in liver metastases in a patient with metastatic PC who had received toripalimab (240 mg Q3W) and gemcitabine (1000 mg/m^2^ on day 1 and 8 every 3 weeks) plus nab-paclitaxel (125 mg/m^2^ on day 1 and 8 every 3 weeks). As a result, he experienced long-term PR (8 cycles at the time of the report) but did not suffer any grade 3 or higher toxicity [38]. A phase Ib/II study in which toripalimab, gemcitabine, and nab-paclitaxel were combined to treat patients with nonresectable PC was initiated by the same center (ChiCTR2000032293, [39]. Additionally, the author is conducting a study using sintilimab + mFOLFIRINOX for metastatic PC patients (NCT03977272), and a study using toripalimab + mFOLFIRINOX for borderline resectable or locally advanced PC patients (NCT03983057). The current ORRs are encouraging and primary endpoints are being followed up.

Tumor cells harness PD-L1 upregulation and antigen processing and presentation defects to evade immunosurveillance. Combining chemotherapies with anti-PD-(L)1 has been predicted to both induce ICD to promote antigen processing and presentation, and block the PD-L1-PD-1 interaction to improve T cell function [29]. However, the presence of rough PC TME, including dense fibrous stroma, high levels of carcinoma-associated fibroblasts (CAFs), MDSCs, and TAMs, impede the delivery and function of systemic therapeutics [40]. Optimization of the scheduling and sequencing of combination therapeutics, investigation of advanced targeting delivery systems, and testing local delivery methods are promising routes for improving the efficacy of therapies and reducing adverse effects (AEs). So far, published clinical studies that combine chemotherapies with anti-PD-(L)1 have been limited by relatively small sample sizes and the lack of comparator arms. The results of randomized controlled trials are anticipated to address the unmet clinical need in PC.

## Combination with radiotherapy or other locoregional therapies

Radiotherapy (RT) is used in PC to sterilize vessel margins, promote margin-negative resection, prevent or delay local progression, palliate pain and bleeding, and relieve obstructive symptoms [41–43]. RT can induce an immune response via promotion of the production of the interferons (IFNs), cytokines, and chemokines, as well as the release of tumor antigens and damage-associated molecular patterns (DAMPs). However, the efficacy of RT alone is insufficient to radically eliminate the tumor. Combining RT with anti-PD-(L)1 may be synergistic for local and distant tumor control [44, 45].

In a preclinical study using a syngeneic orthotopic PC mouse model, the combination of anti-PD-1 antibodies (10 mg/kg) and hypofractionated RT (8 Gy × 3 fractions delivered daily for 3 days, at a dose rate of 3 Gy/minute) improved survival compared with anti-PD-1 or RT alone. The combination resulted in the greatest systemic IFN-γ response and the highest local expression of immune-activation genes, including CD28 and Icos, in all groups [46]. Azad et al. reported that the addition of anti-PD-L1 antibodies (10 mg/kg at day 0, 3, 6 and 9, administered immediately after RT; clone 10F.9G2) to high doses of RT (12 Gy or 5 × 3 Gy delivered daily until the mice were sacrificed) increased the delay in tumor growth in both KPC and Pan02-derived syngeneic subcutaneous PC mouse models. Radiosensitization following PD-L1 blockade was associated with reduced CD11b^+^Gr1^+^ myeloid cell infiltration and enhanced CD45^+^CD8^+^ T cell infiltration. T cell activation markers, including CD69, CD44, and FasL, and the CD8: Treg ratio also increased. The treatment of murine PC cells with RT upregulated PD-L1 expression in a JAK/STAT1-dependent manner [47]. Moreover, it has been reported that ablative RT promotes vessel normalization and enhances the delivery of anti-PD-L1 agents. Using a syngeneic orthotropic UN-KC6141 mouse model of PC, 67% of mice survived more than 30 days after tumor inoculation when using ablative RT (a single dose of 25 Gy) and anti-PD-L1 antibodies (10 mg/kg for 5 days), while the median survival time was 16.5 days within the control group. Furthermore, ablative RT was found to be more effective than conventional fractionated RT at recruiting T cells and combining an anti-PD-L1 agent [48]. Ataxia-telangiectasia mutated (ATM) is an apical kinase responding to ionizing radiation-induced DNA double-strand breaks. In a syngeneic subcutaneous PC mouse model, the efficacy of PD-L1 inhibition (100 μg/mouse Q3W) was demonstrated to be enhanced by the inhibition of ATM (by infecting the cells with shATM virus) and further potentiated by radiation (a single fraction of 8 Gy) with increased tumoral immunogenicity [49].

In a recent phase I/II clinic study, durvalumab (750 mg on day 1 Q2W) and stereotactic RT (6.6 Gy × 5 QOD beginning day 8) were administered to patients with locally advanced or borderline resectable PC. Of the 15 patients recruited to the study, 6 underwent surgery for whom the resections were all margin-negative, while 4 discontinued study treatments due to disease progression. No dose-limiting toxicity (DLT) was identified [50]. A case report has been published in which a patient with locally advanced PC received preoperative radiation (5 Gy × 5) and pembrolizumab (200 mg Q3W) for 4 months. After surgery, a near-complete pathological response was reported [51]. However, in a phase I trial combining pembrolizumab (200 mg Q3W, beginning 1 week before the radiation therapy) with hypofractionated radiotherapy, 4 patients with metastatic PC all displayed progressive disease (PD). Three received 8 Gy × 3 fractions while the other received a 17 Gy × 1 fraction. All RT target lesion sites metastasized within the liver rather than the pancreas [52]. The combination of chemoradiation and anti-PD-(L)1 has also been used clinically. In a randomized phase Ib/II study in which 22 patients with resectable or borderline resectable PC were recruited, 71% of patients that had received pembrolizumab (200 mg Q3W) and capecitabine (825 mg/m^2^ BID, Monday-Friday, on days of radiation only) with radiation (50.4 Gy in 28 fractions over 28 days) underwent surgery, compared with 50% of patients that received only capecitabine and radiation. No grade 4 toxicity was observed in the tri-combination [53].

Although RT reprograms the PC TME to exert a potent antitumor immune response, it may enhance the infiltration of Tregs and MDSCs [44]. The transient accumulation of immunosuppressive CD4^+^ FoxP3^+^ Treg after irradiation was deemed to protect the local tissue against excessive inflammation-induced tissue damage [54]. Preclinical studies have shown that increased radiation dose may in turn weaken immunogenicity [55, 56]. Moreover, while single high-dose irradiation may enhance the immune response, fractionated RT may eliminate this effect [57]. Therefore, the radiotherapy dose, fractionation, in addition to the scheduling of combining anti-PD-(L)1 into the treatment, remain challenging.

Other locoregional therapies have also been investigated in combination with anti-PD-(L)1 agents. Irreversible electroporation (IRE) is a local ablation technique that uses short high-voltage electrical pulses to induce cell death through permanent membrane lysis or loss of homeostasis [58]. The combination of IRE and anti-PD-1 (100 μg per mouse at 30 min after IRE, then QOD for 6 total injections; clone J43) was reported to promote CD8^+^ T cell infiltration and significantly prolong survival in a syngeneic orthotopic PC mouse model, resulting in an immune response that had long-term memory [59]. In a phase Ib trial that treated 10 locally advanced PC patients with surgically-ablative IRE followed by nivolumab (240 mg Q2W), the probability of OS was 67% after 12 months and 33% after 18 months. No DLT was observed. A multicenter phase 2 adjuvant trial using this combination is currently underway [60].

## Combination with molecularly targeted therapy (Fig. 1)

### Combination with agents targeting the extracellular stroma

PC is characterized by a dense fibrous stroma, consisting principally of collagen, hyaluronic acid (HA), and fibronectin [61]. It impedes blood flow, inhibits drug delivery, and suppresses the anti-tumor immune response [62]. In patients with PC, increased HA is associated with decreased survival. Depletion of HA using hyaluronidase diminishes collagen synthesis, depletes chondroitin sulfates, and remodels the stroma of the tumor [63]. In a preclinical model of cancer, the MH194 PC cell line was transfected with hyaluronan synthase-3 (HAS3) to generate a syngeneic PC mouse model with high levels of HA. By combining an anti-PD-L1 antibody (clone 10F.9G2) and PEGylated recombinant hyaluronidase PH20 (PEGPH20, 37.5 μg/kg, the human equivalent dose, 24 h prior to anti-PD-L1), inhibition of the growth of MH194/HAS3 tumors was enhanced (by 79%, *p* < 0.0001 compared with anti-PD-L1 or PEGPH20 alone). Similar findings were obtained when anti-PD-1 (clone RMP1–14) and PEGPH20 were combined (tumor growth inhibition: 56%, *p* = 0.020 and 0.017, respectively, compared with anti-PD-1 or PEGPH20 alone) [64]. The use of PEGPH20 to improve the delivery of anti-PD-1 antibodies and infiltration of cytotoxic T lymphocytes is being investigated in an ongoing clinical trial (3 μg/kg PEGPH20 QW with 200 mg pembrolizumab Q3W) for patients with HA-high refractory metastatic PC [65]. Considering the important role of HA in interstitium formation, AEs of such combination therapies, especially cardiovascular risks, should be closely monitored and evaluated.

**Fig. 1:**
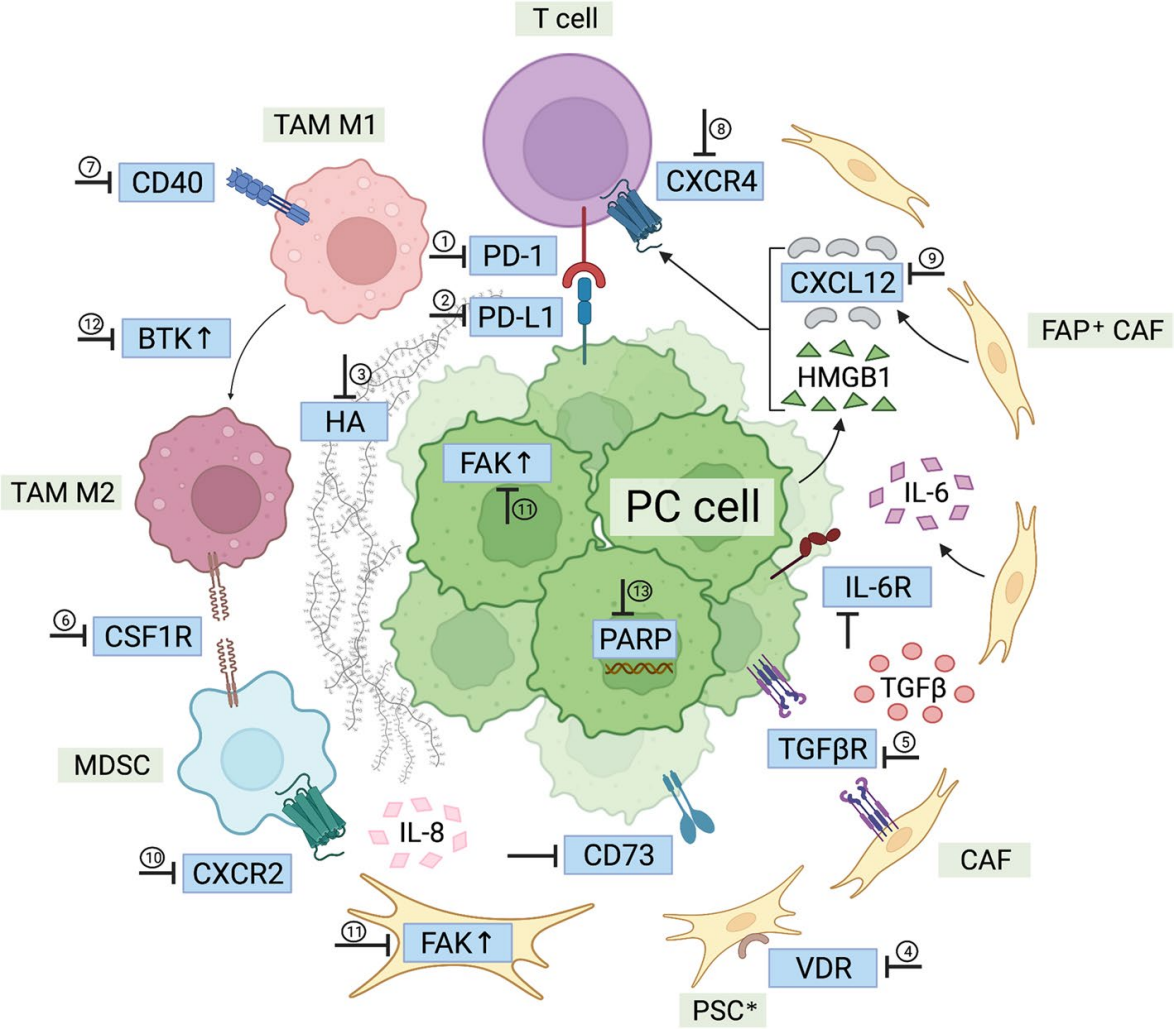
Pancreatic cancer targeted therapies described in the review. ①nivolumab, pembrolizumab, toripalimab; ②durvalumab, avelumab, atezolizumab; ③PEGPH20; ④paricalcitol; ⑤galunisertib, M7824, LY2157299; ⑥cabiralizumab, lacnotuzumab; ⑦APX005M; ⑧BL-8040, AMD3100; ⑨NOX-A12; ⑩SX-682; ⑪defactinib; ⑫acalabrutinib; ⑬niraparib, BGB-290. BTK: Bruton’s tyrosine kinase; CAF: carcinoma-associated fibroblast; CSF1R: colony stimulating factor 1; CXCL12: CXC-chemokine ligand 12; CXCR2: CXC-chemokine receptor 2; CXCR4: CXC-chemokine receptor 4; FAK: focal adhesion kinase; FAP: fibroblast activation protein; HA: hyaluronic acid; HMGB1: high-mobility group protein B1; IL-6R: interleukin-6 receptor; MDSC: myeloid-derived suppressor cell; PARP: Poly (ADP-ribose) polymerase; PC: pancreatic cancer; PD-1: programmed cell death protein 1; PD-L1: programmed cell death ligand 1; PSC: pancreatic stellate cell; PSC*: activated pancreatic stellate cell; TAM: tumor-associated macrophage; TGFβR: transforming growth factor-β receptor; VDR: vitamin D receptor

Interleukin-6 (IL-6) is a cytokine instrumental for the progression of PC and a prognostic factor for outcome. It is produced principally by pancreatic stellate cells (PSCs) but also by tumor-associated myeloid cells [66, 67]. The IL-6/STAT3 axis can promote the expansion of immunosuppressive cells and alter the balance of T cell subsets [68, 69]. In mice bearing subcutaneous MT5 and Panc02 tumors, the combination blockade of anti-IL-6 antibody (200 μg/mouse, TIW for 2 weeks; clone MP5-20F3) and anti-PD-L1 antibody (200 μg/mouse, TIW for 2 weeks; clone 10F.9G2) was found to inhibit tumor volume (*p* < 0.05 compared with monotherapy using either) in CD8^+^ T cell-dependent approaches [70]. In addition, in mice bearing autochthonous KPC-derived pancreatic tumors, mOS increased with the combination of anti-IL-6 and anti-PD-L1 (25 days vs 11 days, *p* = 0.04, compared with mice administered equimolar control antibodies) [71]. While T cell infiltration into tumors is a major issue that anti-PD-(L)1 therapies face, combining anti-IL-6 may switch T cells to a Th1 phenotype and limit immune suppression [70]. Of note, IL-6 blocking antibodies have been approved in clinic to treat autoimmune diseases [72]. Therefore, whether this combination improves PC treatment efficacy still requires clinical verification.

### Combination with agents targeting membrane receptors

PSCs are fibroblasts that become activated and express high levels of vitamin D receptors (VDRs) in the stroma of tumors in PC. Treatment with VDR ligands may convert activated PSCs to a quiescent state, inducing stromal remodeling and reducing tumor volume, thereby permitting greater delivery of chemotherapeutic agents [73]. A clinical trial that combined gemcitabine (1000 mg/m^2^, on day 1, 8, 22, and 29 of a 42-day cycle), albumin-bound paclitaxel (125 mg/m^2^, on day 1, 8, 22, and 29 of a 42-day cycle), cisplatin (25 mg/ m^2^, on day 1, 8, 22, and 29 of a 42-day cycle), nivolumab (240 mg, on day 1, 15, and 29 of a 42-day cycle), and paricalcitol (25 μg BIW) to study the treatment of metastatic PC found that among 24 patients that were evaluated, 19 displayed PR, 2 had stable disease (SD), and 2 exhibited PD. Median PFS was 8.17 months and mOS was 15.3 months. The most common drug-related, grade 3–4 AEs were thrombocytopenia (76%) and anemia (44%). Encouraging ORR values have resulted in an expansion of the trial [74]. Due to the lack of control groups, the role of paricalcitol in improving anti-tumor effects remains unknown. Additionally, considering the relatively heavy chemotherapeutic burden of this combined regimen, AEs require strict prevention and treatment.

Transforming growth factor-β (TGF-β) promotes the expression of fibrous extracellular matrix and enhances tumor immunosuppression, metastasis, and epithelial-to-mesenchymal transition (EMT )[75]. Preclinical trials in other tumors have demonstrated that the combined blockade of TGF-β signaling and PD-(L)1 checkpoint increases CD8^+^ T cell infiltration and reduces immune suppression [75, 76]. Wang and others used acidic tumor extracellular pH-responsive clustered nanoparticles (^LY^iCluster_si*PD-L1*_) to deliver TGF-β receptor inhibitors (LY2157299) and small interfering RNA (siRNA) molecules that targeted PD-L1 in PC. In a Panc02 orthotopic tumor model, tumor weight was found to significantly decline using ^LY^iCluster_si*PD-L1*_ (~ 88% reduction, *p* < 0.001 compared with monotherapy of either). High serum levels of IFN-γ resulted in improved T cell activity [77]. M7824 (MSB0011359C) is a bifunctional fusion protein of anti-PD-L1 antibody fused to the extracellular domain of TGF-β receptor II (TGF-β trap). In a phase I trial of 5 advanced PC patients, one that was mismatch repair-deficient (dMMR) received M7824 at 3 mg/kg and achieved durable PR. The patient persisted in the study and experienced disease progression at 10.5 months. Another PC patient who received M7824 at 3 mg/kg displayed a prolongation of SD for 4.5 months [78]. Moreover, a combination of the TGF-β receptor I kinase inhibitor galunisertib (150 mg BID 14 days on/14 days off) and durvalumab (1500 mg Q4W) was assessed in recurrent or refractory metastatic PC patients in a phase Ib study. One patient experienced PR while 7 displayed SD in a trial of 32 patients, demonstrating a DCR of 25% and mPFS of 1.9 months [79]. Although combined blockade of TGF-β and PD-L1 may display nonredundant effects on the adaptive and innate immune systems, the clinical data were based on heavily pretreated patients and not comparable with chemotherapy.

TAMs and MDSCs actively suppress the anti-tumor immune response, promoting metastatic dissemination and imparting resistance to cytotoxic therapies in human tumors. Colony-stimulating factor 1 (CSF1) signaling regulates their differentiation and survival [80, 81]. While CSF1R blockade was found to upregulate PD-L1 expression, a combination of CSF1R inhibitor (GW2580 800 mg/kg in chow), PD-1 antagonist (200 μg/dose every 4 to 5 days; clone RMP1–14), and gemcitabine (50 mg/kg every 4 to 5 days) reduced tumor progression by more than 90% in a syngeneic orthotopic mouse model [80]. In a phase I study of 31 PC patients that combined the anti-CSF1R antibody cabiralizumab (mostly 4 mg/kg Q2W) and nivolumab (3 mg/kg Q2W), 3 experienced PR (continuing on the study for 293, 275+, and 168+ days) with microsatellite-stable status while 1 displayed prolonged SD (182 days). The 6-month DCR was 13% with an ORR of 10% [82]. A separate study of 30 PC patients that combined the anti-CSF1R antibody lacnotuzumab (MCS110) with the anti-PD-1 antibody spartalizumab (PDR001) resulted in one that experienced PR (continuing in the study for 346 days) and 2 that displayed durable SD (on the study for 328 and 319 days) [83]. Although CSF1 signaling can regulate both the number and the function of TAMs, the PC immunosuppressive microenvironment involves a complex regulatory network, which limits the efficacy of the combined regimen.

CD40 is a member of the tumor necrosis factor (TNF) receptor superfamily that allows dendritic cells (DCs) to promote antitumor T cell activation and re-educates macrophages to destroy the tumor stroma. Agonistic anti-CD40 antibodies and trimeric CD40 ligands were studied to determine whether they activated DCs and other APCs [84]. Using an orthotopic Pan02 tumor mouse model, combining the CD40 agonist (3 mg/kg on day 7, 14, 21, 28; clone FGK4.5) with PD-L1 blockade (10 mg/kg on day 7, 10, 14, 17, 21, 24; clone 10F.9G2) improved OS versus monotherapy with either agent. PD-L1 upregulation, systemic APC maturation, and memory T cell expansion were demonstrated after the use of a CD40 agonist [85]. Furthermore, in a syngeneic subcutaneous PC mouse model, the addition of anti-PD-1 antibodies (200 μg on day 0, 3, 6, 9, 12, 15, 18, and 21; clone RMP1–14) to the CD40 agonist (100 μg on day 3; clone FGK45) and gemcitabine (120 mg/kg on day 1) + nab-paclitaxel (120 mg/kg on day 1) doubled long-term survival compared with a CD40 agonist + gemcitabine + nab-paclitaxel (26% versus 12%). Furthermore, the addition of a cytotoxic T-lymphocyte antigen-4 (CTLA-4) antibody (200 μg on day 0, 3, and 6; clone 9H10) more than trebled long-term survival (39% versus 12%) [86]. In another study in which a genetically engineered PC mouse model was used, the combination of CD40 agonist (100 μg on day 11; clone FGK4.5), radiotherapy (20 Gy on day 8), anti-PD-1 antibodies (200 μg on day 5, 8, 11; clone RMP1–14), and anti-CTLA-4 antibodies (200 μg on day 5, 8, and 11; clone 9H10) eradicated primary and abscopal tumors, thereby providing long-term immunity. While radiotherapy triggers an early proinflammatory response and upregulates MHC class I and CD86 antigens on APCs, CD40 agonists prompt systemic and intratumoral myeloid compartment reorganization. Subsequent immune checkpoint blockade increases intratumoral T cell infiltration and increases the CD8 T cell: Treg ratio. This observation demonstrates that CD40 agonist and radiotherapy can functionally augment PC antitumor immunity [87]. In a clinical phase Ib study in which CD40 agonist APX005M (sotigalimab, 0.1 mg/kg or 0.3 mg/kg on day 3 or day 10), gemcitabine (1000 mg/m^2^ on day 1, 8, and 15 every 4 weeks), and nab-paclitaxel (125 mg/m^2^ on day 1, 8, and 15 every 4 weeks) were combined with nivolumab (240 mg on day 1 and 15 every 4 weeks) to treat 12 metastatic PC patients, 8 achieved PR while 3 displayed SD. Twenty one-day chemotherapy cycle and dose reduction (except for nivolumab) to manage toxicity were allowed. Median PFS was 10.8 months (in the cohort receiving 0.1 mg/kg APX005M) and 12.4 months (in the cohort receiving 0.3 mg/kg APX005M). It was established that the combination was tolerable and later-phase trials are anticipated [88]. Unlike blocking tumor progression or invasion signals in part, CD40 agonism is an “ignition” signal resulting in APC activation, macrophage tumoricidal activity, and CD8^+^ T cell-dependent antitumor immunity. Therefore, administration of a CD40 agonist several days after chemotherapy or radiotherapy may amplify the tumor antigen-induced immune responses. The development of novel drug delivery systems in PC may further improve efficacy.

To counter anti-tumor immunity, PC cells release high-mobility group protein B1 (HMGB1), which combines with CXC-chemokine ligand 12 (CXCL12) produced by fibroblast activation protein (FAP)-positive CAFs to activate CXC-chemokine receptor 4 (CXCR4) on Teffs [89]. In a syngeneic subcutaneous PC mouse model, the addition of anti-PD-L1 antibodies (160 μg QOD; clone 10F.9G2) to 90 mg/mL CXCR4 inhibitor AMD3100 (using an AZLET osmotic pump) was found to significantly decrease tumor volume (*p* < 0.01), identified by the loss of heterozygosity of the Trp53 gene [90]. In a study in which human PC specimens were harvested and analyzed by organotypic slice culture to test the effects of the combination of anti-PD-1 (20 mg/mL) and CXCR4 inhibitor AMD3100 (100 mg/mL), CD8^+^ T cell migration and induction of tumor cell apoptosis were observed [91]. In a Panc02 subcutaneous tumor mouse model, CXCR4 antagonist BL-8040 combined with anti-PD-1 and irinotecan, fluorouracil and leucovorin reduced tumor growth by 58% compared with chemotherapy alone. Although the triple combination treatment did not significantly change the number of CD8^+^ T cells in the tumor, it increased their activation state [92]. In a clinical study, administration of the CXCL12 inhibitor NOX-A12 and pembrolizumab resulted in 25% of patients experiencing SD in 11 metastatic colorectal cancer patients and 9 metastatic PC patients. Median PFS was 1.87 months, OS was 42% after 6 months and 22% after 12 months [93]. A similar DCR of 21.4% (1 PR + 2 SD) was achieved using BL-8040 (1.25 mg/kg for 2 weeks before pembrolizumab and on day 1, 4, 8, 11 combined with pembrolizumab in a 3-week cycle) and pembrolizumab (200 mg Q3W) in metastatic PC patients in a similar study [94]. Furthermore, in a phase IIa two-cohort study, 22 metastatic PC patients received BL-8040 (1.25 mg/kg on day 1–5 of week 1, then 1.25 mg/kg BIW from week 2), pembrolizumab (200 mg Q3W from day 10), liposomal irinotecan (70 mg/m^2^ Q2W from day 8), fluorouracil (2400 mg/m^2^ Q2W from day 8), and leucovorin (400 mg/m^2^ Q2W from day 8), finding that the ORR, DCR, and the median duration of response were 32, 77%, and 7.8 months, respectively. In a separate cohort, 37 patients with chemotherapy-resistant stage II-IV PC received BL-8040 (1.25 mg/kg on day 1–5 of week 1, then 1.25 mg/kg TIW from week 2) and pembrolizumab (200 mg Q3W). The investigators found a DCR of 34.5% in the study population with an mOS of 7.5 months in patients receiving the treatment as second-line therapy [95]. Blocking the CXCL12/CXCR4 pathway attempts to address the problem of geographic sequestration of Teffs, which emphasizes the sequestration of clonally expanded Teffs from the juxtatumoral compartment [91]. Combined with anti-PD-(L)1, it modulates different axes of immunosuppression in PC that demonstrates a synergistic effect.

CXCR2 is a G-protein-coupled receptor that regulates neutrophil and MDSC migration in the TME of PC, which drives tumor invasion and metastasis. In an experiment using KPC mice, one group firstly received the CXCR2 inhibitor AZ13381758 100 mg/kg BID for 2 weeks to increase T cell infiltration into the tumor, followed by the administration of anti-PD-1 antibodies (10 mg/kg BIW) while AZ13381758 continued. They experienced survival that was significantly extended compared with mice treated with vehicle plus anti-PD-1 [96]. In addition, using the KP2 orthotopic PC mouse model, triple therapy with the CXCR1/2 inhibitor SX-682, FOLFIRINOX, and checkpoint inhibition (anti-PD-1/anti-CTLA-4) resulted in significantly increased survival compared with other groups [97]. Considering that CXCR2 is principally upregulated in neutrophils and MDSCs rather than in cancer cells, its inhibition in combination with anti-PD-(L)1 may be used with standard chemotherapy to improve efficacy.

CD73 is an ectoenzyme that catalyzes the hydrolysis of adenosine monophosphate (AMP) to immune-suppressive adenosine (ADO). It curtails T cell activation and is associated with a worsening prognosis in malignancies. In C57BL/6 J mice bearing established KP4662-G12C tumors, coadministration of CD73 inhibitor A0001421 (30 mg/kg QD) and anti-PD-1 antibodies (10 mg/kg BIW; clone RMP1–14) was superior to anti-PD-1 alone in limiting PC growth [98]. KRAS mutation is one of the most common oncogenic drivers in PC, which significantly upregulates CD73 expression and results in a worsening prognosis. In addition, PD-1 nonresponsive mice express higher levels of ADO pathway genes, including CD73 and CD39. Therefore, inhibiting CD73 together with PD-(L)1 is hoped to provide a significant anti-tumor effect, especially in PC [98–100].

### Combination with agents targeting intracellular regulators

Focal adhesion kinases (FAKs, including FAK1 and PYK2) are non-receptor tyrosine kinases that translate signals from the extracellular matrix into intracellular pro-inflammatory pathway regulation and cytokine production. Wound healing and pathological fibrosis both involve FAK signaling [101, 102]. FAK activity is elevated in human PC tissue and correlates with increased fibrosis and poor CD8^+^ cytotoxic T cell infiltration. Using both syngeneic and genetic animal models, FAK inhibitor VS-4718 (50 mg/kg BID) was found to promote responsiveness to a PD-1 antagonist (200 μg every 4–5 days; clone RMP1–14), demonstrated by reduced tumor burden and improved OS. The maximum response was observed when FAK inhibition was provided in combination with low-dose gemcitabine (25 mg/kg every 4–5 days) [102]. In a phase I study that combined the FAK inhibitor defactinib, pembrolizumab, and gemcitabine, PR was observed in a patient who had progressed when treated with gemcitabine and nab-paclitaxel. The median duration of patient treatment was 127 days in a cohort of 8 PC patients, the longest duration being 290 days. The combination regimen was found to be well-tolerated, and recruitment of additional patients to the cohort (PC only) is ongoing [103]. Considering the immunosuppressive TME and the uniquely desmoplastic stroma, FAK inhibition is particularly promising in PC. However, FAK inhibition and anti-PD-(L)1 both have strong molecule-targeting capabilities and general killing effects, and so still require treatment based on chemotherapy.

Bruton’s tyrosine kinase (BTK) is a non-receptor enzyme of the Tec kinase family expressed by B cells and macrophages. In a murine model of PC, BTK inhibitor ibrutinib was shown to convert M2-like macrophages to an M1-like phenotype, resulting in the promotion of CD8-mediated T cell cytotoxicity [104]. A phase II study evaluated the BTK inhibitor acalabrutinib (100 mg BID) alone or in combination with pembrolizumab (200 mg Q3W) in 77 advanced PC patients. The ORR and DCR values of 0 and 14.3% were observed with monotherapy, and 7.9 and 21.1% for combination therapy. An exceptional responder treated with this combination therapy experienced a PR with near-resolution of bulky peritoneal disease. An additional exceptional responder that had initially been prescribed acalabrutinib alone exhibited SD for 7.5 months, and on crossover demonstrated SD in response to combination therapy, remaining on combination therapy for 8.7 months before progression. The combination of acalabrutinib and pembrolizumab was well tolerated but demonstrated limited clinical effectiveness [105]. The enrolled patients had failed a median of 3 prior lines of therapy, possibly leading to a clonal expansion of a kinase inhibitor-resistant phenotype likely to eliminate the oncogenic drivers responsive to BTK inhibition [106]. For this kinase-targeted therapy, the difficulty of patient selection limits its clinical efficacy.

Poly (ADP-ribose) polymerase (PARP) recognizes and binds to single-stranded DNA breaks, and recruits proteins to perform DNA damage repair (DDR). PARP inhibitors prevent DDR, probably via trapping PARP on the DNA and preventing the progression of the replication fork. Cancer cells with mutations that prevent homologous recombination repair via other pathways, such as loss-of-function mutations in BRCA1/2, are often more sensitive to PARP inhibitors because of the substantially reduced capacity for DDR [6]. A study in which PARP 1/2 inhibitor BGB-290 was combined with the anti-PD-1 antibody BGB-A317 to treat advanced PC patients found that 1 subject experienced PR while 2 achieved SD. They had received BGB-290 + BGB-A317 for 189 and 281 days, respectively [107]. Furthermore, SD in PC patients was also observed when the PD-1 inhibitor dostarlimab, the PARP inhibitor niraparib, and bevacizumab were administered as a combination therapy in another study [108]. Because of the lack of control groups, whether the patients who did not derive benefit or were resistant to either PARP inhibition or anti-PD-(L)1 alone could have benefitted from the combination strategy is unknown. In addition, the underlying biological mechanisms of the combination strategy await elucidation to determine whether the agents should be restricted to DDR tumors or used more broadly.

## Combination with immunotherapy

### Combination with agents targeting other immune checkpoints

PD-(L)1 blockade and anti-CTLA-4 therapy operate in different phases of the immune response, which explains the basis of their combined mechanism of action. PD-1 is mostly expressed on late effector phase CD4^+^ helper T cells and CD8^+^ cytotoxic T cells in peripheral tissues. Anti-PD-1 treatment targets the effector phase of T cell activation in this periphery. CTLA-4 is among the first negative regulators to be induced and directly competes with CD28 for the ligands CD80 and CD86, thereby interfering with positive costimulatory signals. Anti-CTLA-4 treatments mostly result in changes in secondary lymphoid organs during the initial phase of naive T cell activation [16, 17, 109, 110]. In a clinical study of 3 refractory PC patients that received nivolumab and ipilimumab (ipilimumab 1 mg/kg and nivolumab 3 mg/kg Q3W followed by nivolumab 240 mg Q2W), 1 patient expressing BRCA1 achieved CR and thereafter received nivolumab on maintenance for 17 months. A second patient that expressed RAD51C achieved PR but continued to improve in terms of pain and tumor markers [111]. A phase II study in which nivolumab (240 mg Q2W) and ipilimumab (1 mg/kg Q6W) were combined with radiation (8 Gy × 3 at cycle 2 QOD) to treat metastatic microsatellite stable PC patients, found a DCR of 20% (5/25) with an ORR of 13% (3/25) via intention to treat analysis [112]. The addition of durvalumab to tremelimumab (durvalumab 1.5 g + tremelimumab 75 mg Q4W × 4 doses → durvalumab 1.5 g Q4W) for the treatment of metastatic PC patients found that more ≥ grade 3 treatment-related AEs were observed (22% with the combination therapy, 6% with durvalumab alone), while mPFS (both 1.5 months) and mOS (3.1 months in combination, 3.6 months for durvalumab alone) did not change [113]. A phase II study combined gemcitabine (1000 mg/m^2^ on day 1, 8, 15 every 4 weeks), nab-paclitaxel (125 mg/m^2^ on day 1, 8, 15 every 4 weeks), durvalumab (1500 mg on day 1 Q4W), and tremelimumab (75 mg on day 1 Q4W) as the first-line therapy to treat metastatic PC patients. A total of 8/11 patients (73%) achieved PR with a DCR of 100%. Median PFS was 7.9 months while the 6-month survival rate was 80% [114]. High-dimensional single-cell profiling was used to profile the response of T cell populations to anti-PD-(L)1 monotherapy, anti-CTLA-4 monotherapy, or their combination. It was shown that the majority of the effects of monotherapies are additive in the context of combination therapy, while combination therapy mediates a switch from an expansion of phenotypically exhausted CD8 T cells to an expansion of activated effector CD8 T cells [115]. However, based on the results of clinical studies, chemotherapy remains the cornerstone of this immune checkpoint inhibitor (ICI) combination strategy.

The immune checkpoint target indoleamine 2,3-dioxygenase 1 (IDO1) is a rate-limiting metabolic enzyme that converts tryptophan into downstream kynurenines. IDO1 is activated in a variety of human cancers and demonstrates immunosuppression through the suppression of CD8^+^ Teffs and NKs, in addition to increased activity of Tregs and MDSCs [116, 117]. A phase I study in Japan combined the IDO1 inhibitor epacadostat (INCB024360, 25 mg or 100 mg BID) with pembrolizumab (200 mg Q3W) to treat advanced PC tumors. One patient achieved PR and remained on treatment for 21 weeks [118]. In another phase I study, one PC patient that received the IDO1 inhibitor navoximod (GDC-0919, 100 mg Q12H) and atezolizumab (1200 mg Q3W) experienced PR. The patient remained in the study for more than 650 days [119]. Based on the clinical results of adding IDO1 inhibition to anti-PD-(L)1 in various cancers, the purpose of improving clinical efficacy in unselected patients has not been achieved.

### Combination with CAR-T therapy

Chimeric antigen receptor (CAR) T cells recognize tumor-associated antigens in an MHC-restricted manner, thereby becoming activated to kill cancer cells. CARs usually consist of an extracellular ligand recognition domain fused to the cytoplasmic domains of one or more costimulatory domains (such as CD28 or 4-1BB) and CD3 zeta [120, 121]. A CAR comprising the extracellular domain of PD-1 not only recognizes the ligands of PD-1 expressed on cancer cells, but also interferes in the interactions of PD-1/PD-L1 that attenuate immune inhibition. In a subcutaneous Pan02 tumor mouse model, chimeric PD-1 T cells were found to significantly reduce tumor burden and increase survival compared with wild-type PD-1 T cells [122]. Another research group developed both PD1ACR (involving a PD-1-CD28–4-1BB activating chimeric receptor) T cells and PDL1CAR T cells using third-generation CARs. In syngeneic PC models, the adoptive transfer of both enhanced T cell persistence and induced CFPAC1 cancer regression by > 80% [123]. Distinct from monoclonal antibody therapies, although CAR T cells have not persisted over the long-term in tumor-surviving mice, they induced a long-lived tumor-antigen-specific host memory responses which protected against the rechallenge of the same tumor. It is worth noting that the host immune response was specific for tumor antigens instead of PD-1 ligands [122, 123]. Moreover, clinical trials investigating CAR T cells targeting mesothelin, carcinoembryonic antigen, mucin 1, and human epidermal growth factor receptor 2 found mixed efficacy in PC [124–128]. These once again demonstrated that a fundamental issue with CAR T cell therapy in PC is the lack of an ideal single-antigen target, which requires further investigation.

### Combination with cancer vaccines

The combination of cancer vaccines and ICIs is likened to revving an engine and releasing the brakes. Cancer vaccines induce the infiltration of Teffs into tumors and create immune checkpoint signals. ICIs further attenuate tumor immune suppression [129]. Cancer vaccines include DC vaccines, whole-cell vaccines, peptide-based vaccines, mRNA vaccines, and DNA vaccines (plasmid vaccines, virus-based vaccines, bacterial vectors, and yeast-based recombination vaccines). DCs are a key member of the family of APCs, which efficiently present antigens to CD4^+^ and CD8^+^ T cells in addition to secreting cytokines such as IL-12, IL-15, IFN-γ, and TNF. DC vaccines can be activated by tumor antigens and promote a cytotoxic T cell response. Blocking PD-L1 on DC may improve the efficacy of DC therapy [130–132]. A recent study used antigen-primed monocyte-derived dendritic cells (MoDCs) alone or in combination with PD-L1 blockade to treat metastatic PC tumors. PD-L1 blockade was performed by adding soluble CD80 or anti-PD-L1 to MoDCs. As a result, 5/10 patients who did not respond to MoDC therapy alone achieved secondary stabilization (4 to 8 months) by the use of MoDCs modified using PD-L1 blockade [132]. Additionally, the same group studied the safety and efficacy of combining antigen-primed MoDCs with nivolumab (1–2 mg/kg 1 day before the DC vaccine). Two of 7 metastatic PC patients achieved PR. The majority of patients tolerated the therapy well with only mild AEs following therapy with nivolumab [133]. Furthermore, a phase Ib study combined a personalized autologous DC vaccine, standard of care adjuvant chemotherapy followed by nivolumab in 3 resectable PC patients. The personalized neoantigen peptides were identified through a proteo-genomics antigen discovery pipeline for each patient. The study described optimal conditions for incorporation into long peptides for manufacture into vaccine products [134]. It was shown in the preclinical study that DCs loaded with a PD-L1 immunogen could induce anti-PD-L1 antibodies and a T cell response in vivo. The PD-L1-specific cytotoxic T cells displayed cytolytic activity against PD-L1^+^ tumor cells. This provided a novel concept for combining DC vaccines and anti-PD-(L)1. Without the repeated administration of anti-PD-(L)1 antibodies, this novel PD-L1 vaccination persistently produced anti-PD-L1 antibodies and T cell responses. This strategy may benefit not only cancer treatment but immune prevention of carcinogenesis in healthy individuals at high risk of developing cancer, in addition to the prevention of cancer recurrence [135].

GVAX is a human whole-cell granulocyte-macrophage colony-stimulating factor (GM-CSF)-secreting PC vaccine, consisting of allogeneic PC cell lines engineered to secrete the factor. It significantly upregulated the expression of PD-L1 on the membrane following treatment of tumor-bearing mice. In a syngeneic metastatic PC mouse model, combination therapy with GVAX (on day 4, 7, 14, and 21) and anti-PD-1 G4 antibodies (100 μg BIW) improved murine survival compared with anti-PD-1 antibody monotherapy or GVAX therapy alone [136]. The addition of anti-CD137 antibodies (20 μg BIW for 4 weeks; clone BMS-469452) to GVAX (on day 4, 7, 14, and 21) and anti-PD-1 antibodies (100 μg BIW for 4 weeks; clone 4H2) further improved survival in a syngeneic liver-metastatic PC mouse model. The expression of the costimulatory molecules CD137 and OX40 on CD4^+^PD-1^+^ and CD8^+^PD-1^+^ T cells increased, suggesting activation of the T cells [137]. Moreover, the addition of the CD40 agonistic antibody to GVAX and anti-PD-1 also increased the survival of mice with syngeneic metastatic PC [138]. In terms of the dose and schedule for anti-PD-(L)1 and vaccines, it has been shown that both PD-1 and PD-L1 appear rapidly following exposure to IFN, which emphasizes the early application of PD-(L)1 blockade [134].

### Combination with oncolytic virus

Oncolytic virus (OV) immunotherapy involves three major mechanisms: direct infection of cancer cells, endothelial cells, and subsequent oncolysis; indirect effects of necrosis or apoptosis of uninfected cancer cells and their associated cells within the TME; improved antigen cross-priming and recruitment of immune cells into the TME. When combined with ICIs, OVs prime, boost, and recruit Teffs into the TME, where ICIs enhance the potency of lymphocytes to infiltrate tumors via the removal of inhibitory signals [139, 140]. In a preclinical study using a KPC syngeneic PC mouse model, Wang et al. found that oVV-LZ8 (where the oVV-gene is an oncolytic vaccinia virus platform and LZ8 is an immunomodulatory protein isolated from Reishi, capable of promoting cell proliferation and IL-2 production in T cells) enhanced the tumor inhibitory effects of anti-PD-1 antibody in vivo. It was associated with increased numbers of NK, CD8^+^, and CD4^+^ T cells in addition to increased secretion of anti-tumor cytokines, including IFN-α, IFN-γ, and IL-2 [141]. VVL-21 is an oncolytic vaccinia virus carrying IL-21, a regimen that inhibits PI3Kδ to prevent viral uptake by macrophages so that intravenous delivery is enhanced, with viral B5R protein modification to improve viral spread within and between tumors. By combining VVL-21 with anti-PD-1, tumor growth was found to be significantly inhibited compared with the virus alone in a syngeneic subcutaneous DT6606 tumor mouse model. Enhanced IFN-γ induction and a more significant CD8^+^ T cell response resulted in more greatly increased OS [142]. Moreover, CF33-hNIS-antiPDL1 is a genetically engineered chimeric orthopoxvirus equipped with human sodium iodide symporter (hNIS) and anti-PD-L1 antibody. In a syngeneic peritoneal carcinomatosis nude mouse model of PC, a single dose of CF33-hNIS-antiPDL1 produced functional anti-PD-L1 antibody, reduced tumor burden, and prolonged survival [143].

In a clinical study, the combination of the oncolytic reovirus pelareorep with pembrolizumab and chemotherapy resulted in 1 PC patient achieving PR for 17.4 months, while 2 experienced SD for 9 and 4 months. Viral replication was observed in biopsies of the tumor. High peripheral T cell clonality and changes in the expression of immune genes were observed in patients, resulting in clinical benefit. The combination therapy did not display any significant toxicity [144].

The immunosuppressive PC TME poses a double-edge sword in OV immunotherapy. It helps with viral replication that promotes direct oncolysis, but also impedes the following antitumor immune responses and limits durable immunity. Methods to regulate the TME to manipulate this balance may determine the optimal clinical efficacy of OV. Moreover, the mechanisms of primary, adaptive, and acquired resistance to OV immunotherapy remain to be elucidated. Combining anti-PD-(L)1 with OV may partly overcome the resistance but has not demonstrated a radical improvement. Drugs regulating the TME can be either physically delivered or encoded by a recombinant OV to access the tumor to maximize the efficacy of this combination.

## Discussion

Anti-PD-(L)1 monotherapy has not achieved satisfactory clinical outcomes in PC, due to the immunosuppressive TME and its intrinsically non-immunogenic nature. Mechanisms to evoke the response of PC to ICIs include increasing initial T cell priming, neutralizing immunosuppressive elements within the TME, enhancing innate and other adaptive immune cell effector functions, and inhibition of compensatory T cell anergy and exhaustion [109, 145]. Various systemic and locoregional therapies have been investigated for combination with anti-PD-(L)1 therapy founded on this concept, but the efficacy and AEs of these combinations vary widely.

In terms of mechanisms, targeted therapies have strong specificity, while their anti-tumor effects may be mediocre. It may be due to the multi-faceted roles of the drug targets, which probably regulate the PC TME through a variety of cell types and pathways that do not simply suppress the tumor. Immunotherapies are highly dependent on the specific PC TME, which is complex for different tumors, displaying various responses. Chemotherapies powerfully induce tumor cell death, release tumor-associated antigens, and initiate a following immune response. However, chemotherapy alone tends to induce drug resistance, which leads to a subsequent poor response to other types of anti-tumor treatment.

In preclinical studies, cell and mouse models inadequately recapitulate the whole genomic heterogeneity and the TME of human PC. In particular, syngeneic subcutaneous PC mouse models are mostly used in current preclinical studies, but are relatively deficient in the host immune responses and have low disease complexity. Using humanized mouse models can help elevate the validity of evidence [146, 147]. In addition, animal samples are often limited to a small size. The dose, schedule, and sequence of drug administration vary greatly across different preclinical studies, resulting in significant differences in efficacy and survival. Moreover, the monitoring of AEs in animals is relatively scarce, restricting toxic dose limitation and dose adjustment.

In clinical studies, a number of researchers have selected patients based on predictive biomarkers or genetic profiling, while others have not investigated specific molecular characteristics. Some have excluded patients who had used the same types of drug, while others have not. The treatment lines of different combinations also vary widely. This has resulted in significant heterogeneity among enrolled patients. Focusing on a subgroup that did not derive benefit or was resistant to either monotherapy may be challenging but have meaningful conclusions. Moreover, some clinical studies are single-arm trials without control groups. The dosing and scheduling of drugs differ greatly across studies, as determined by preclinical protocols or clinical experience, and still require adjustments and optimization based on sequential tumor biopsies and serial blood collection.

A notable problem with combination therapies is whether AEs exceed acceptable clinical limits. When combining pembrolizumab (2 mg/kg Q3W) with gemcitabine (1000 mg/m^2^ on day 1 and 8 every 3 weeks) and nab-paclitaxel (125 mg/m^2^ on day 1 and 8 every 3 weeks) for the treatment of PC, 70.6% of patients experience grade 3–4 treatment-emergent adverse events (TEAEs), which declines following mandatory premedication with dexamethasone [36]. A separate study that tested nivolumab (3 mg/kg on day 1 and 15 every 4 weeks) + gemcitabine (1000 mg/m^2^ on day 1, 8, 15 every 4 weeks) and nab-paclitaxel (125 mg/m^2^ on day 1, 8, 15 every 4 weeks) reported that 98% of patients either experienced ≥1 grade 3–4 TEAE (96%) or discontinued treatment due to TEAEs (36%). That study deemed that the drugs were tolerable but the observed efficacy did not support further investigation. The combination was not characterized by additional immune-related AEs, and the reported AEs were consistent with those of each agent [37]. The tri-combination of pembrolizumab (200 mg Q3W), capecitabine (825 mg/m^2^ BID, Monday-Friday, on days of radiation therapy only), and radiation (50.4 Gy in 28 fractions over 28 days) treatment was relatively well-tolerated with no grade 4 toxicity or major surgical complications 30 days postoperatively [53]. As for AEs due to combining molecularly targeted agents with anti-PD-(L)1, dose-escalation of the TGF-β receptor I kinase inhibitor galunisertib (150 mg BID 14 days on/14 days off) did not display DLTs, at the maximum dose of 150 mg BID that was selected. Adding it to durvalumab (1500 mg Q4W) led to no more than 7 grade ≥ 3 related AEs in 32 PC patients [79]. When combining the anti-CSF1 antibody lacnotuzumab with spartalizumab, grade ≥ 3 AEs increased, such as elevated aspartate aminotransferase (12%), asthenia (10%), and hyponatremia (10%) [83]. The combination of sotigalimab (CD40 agonistic antibody, 0.1 mg/kg or 0.3 mg/kg on day 3 or day 10), gemcitabine (1000 mg/m^2^ on day 1, 8, and 15 every 4 weeks) plus nab-paclitaxel (125 mg/m^2^ on day 1, 8, and 15 every 4 weeks), and nivolumab (240 mg on day 1 and 15 every 4 weeks) was found to cause grade 3 or 4 treatment-related AEs in 94% of patients, but which were clinically manageable. One death due to septic shock occurred due to neutropenia. Toxicity was deemed chemotherapy-related and synergistic toxicity was not apparent when combining sotigalimab with nivolumab [88]. Most common grade ≥ 3 AEs using gemcitabine (1000 mg/m^2^ on day 1, 8, 15 every 4 weeks), nab-paclitaxel (125 mg/m^2^ on day 1, 8, 15 every 4 weeks), durvalumab (1500 mg on day 1 Q4W), and tremelimumab (75 mg on day 1 Q4W) were hypoalbuminemia (45%), abnormal lipase (45%), and anemia (36%) [114]. Combining anti-PD-(L)1 with anti-CTLA-4 was found to increase, predate, and raise the severity of AEs in various cancers [148, 149]. The use of the oncolytic reovirus pelareorep with pembrolizumab and chemotherapy was observed to cause grade 3–4 treatment-related AEs in 18% of patients [144]. Thus, the occurrence of AEs when combining anti-PD-(L)1 with different agents is variable but closely associated with drug dosage. Whether there is greater risk and earlier onset of AEs requires practical analysis. Systemic glucocorticoids should be used with caution. Clinicians and scientists need to perform a baseline evaluation before treatment, a comprehensive examination during treatment, a close follow-up after treatment, in addition to an individualized treatment [150].

## Future perspectives

**Fig. 2:**
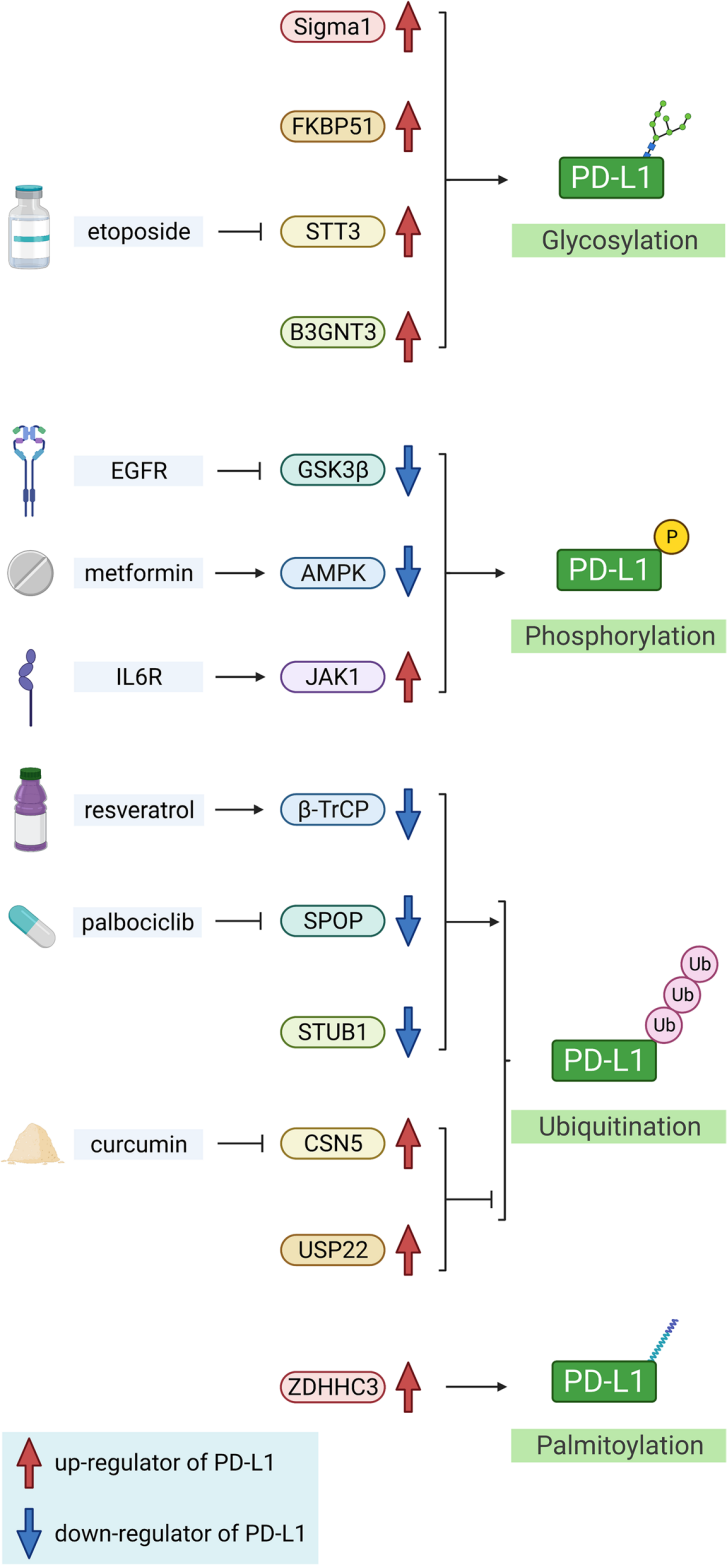
Post-translational modifications of PD-L1 described in the review. AMPK: adenosine-5′-monophosphate-activated protein kinase; B3GNT3: β-1,3-N-acetylglucosaminyl transferase; CSN5: COP9 signalosome complex subunit 5; EGFR: epidermal growth factor receptor; FKBP51: FK506 binding protein 51; GSK3β: glycogen synthase kinase 3β; IL6R: interleukin-6 receptor; JAK1: Janus kinase 1; PD-L1: programmed cell death ligand 1; Sigma1: sigma nonopioid intracellular receptor 1; SPOP: speckle-type POZ protein; STT3: staurosporine temperature sensitivity 3; STUB1: STIP1 homology and U-box containing protein 1; USP22: ubiquitin-specific protease 22; ZDHHC3: zinc-finger DHHC-type-containing 3; β-TrCP: β-transducin repeat-containing protein

In addition to the challenging discovery and development of new drugs, drug repurposing has been recognized as an attractive investigation proposition, with fewer development costs, shorter development timelines, and lower safety risks [151]. With the focusing of research on the post-translational modifications (PTMs) of PD-(L)1, various traditional chemotherapeutic and targeted drugs have been found to regulate the PTMs of PD-(L)1, thus demonstrating the prospect of combining with anti-PD-(L)1. Gemcitabine and paclitaxel have been shown to induce PD-L1 expression via the JAK/STAT pathway, and attenuated by a JAK2 inhibitor [31]. The epidermal growth factor (EGF) receptor tyrosine kinase inhibitor gefitinib phosphorylates PD-L1 to induce its degradation. Gefitinib has been combined with PD-1 blockade, displaying enhanced therapeutic efficacy in a syngeneic mouse cancer model [152]. Anti-IL-6 inhibits JAK1 activation to promote the degradation of PD-L1, when combined with an ICI, demonstrated enhanced anti-tumor efficacy compared with the ICI alone [153]. The PTMs of PD-(L)1 restrict the synergistic effects of combination therapies mentioned above. However, combination therapies with drugs targeting the PTMs of PD-L1 have scarcely been studied for PC, although these have emerged as potential strategies for cancer treatments over recent years (Fig. 2).

### Targeting the PTMs of PD-L1

The PTMs of PD-L1 are important mechanisms that regulate protein stability, translocation, and protein-protein interactions. Glycosylation, phosphorylation, ubiquitination, and palmitoylation are the four major PTMs of PD-L1. N-linked glycosylation protects PD-L1 from glycogen synthase kinase 3β (GSK3β)-mediated 26S proteasome machinery engagement [152, 154]. It also enriches PD-L1 in cancer stem cells and regulates PD-L1/PD-1 interaction, thus suppressing the T cell anti-tumor response [155, 156]. Chaperone sigma nonopioid intracellular receptor 1 (Sigma1) and cochaperone FK506 binding protein 51 (FKBP51) facilitate PD-L1 glycosylation and stabilization in the endoplasmic reticulum (ER). Inhibition of Sigma1 or FKBP51 has been demonstrated to reduce PD-L1 expression and activate T cells in vitro in prostate cancer, triple-negative breast cancer (TNBC), and glioblastoma models [157, 158]. Staurosporine temperature sensitivity 3 (STT3) is the catalytically active subunit of oligosaccharyltransferase in cancer stem cells that increases PD-L1 glycosylation. Antagonism of STT3 by etoposide has been shown to destabilize PD-L1 and sensitize cancer cells to T cell immunoglobulin mucin-3 (TIM3) antagonists in syngeneic mouse models of TNBC and colon cancer [155]. β-1,3-N-acetylglucosaminyl transferase (B3GNT3)-mediated poly-N-acetyllactosamine is required for the interaction between PD-L1 and PD-1. Anti-glycosylated PD-L1 antibodies induce PD-L1 internalization and degradation in lysosomes, based on which a new antibody-drug conjugate (anti–gPD-L1-MMAE) has been formed that helps eradicate TNBC cells [156, 159].

Phosphorylation regulates the conformation, activity, and interactions of proteins. GSK3β is a serine/threonine kinase that phosphorylates PD-L1’s extracellular domain, which promotes the interaction of PD-L1 with E3 ligase β-transducin repeat-containing protein (β-TrCP) and subsequent PD-L1 degradation [152]. The inhibition of EGF signaling, which is upstream of GSK3β, downregulates PD-L1 and enhances the therapeutic efficacy of PD-1 blockade in syngeneic mouse cancer models [152, 160]. Adenosine-5′-monophosphate-activated protein kinase (AMPK) is an additional serine/threonine kinase that phosphorylates PD-L1 to induce abnormal PD-L1 glycosylation and its ER-associated degradation (ERAD). The AMPK agonist metformin facilitates the ERAD of PD-L1 that promotes antitumor immunity [161]. Moreover, IL-6/JAK1 phosphorylates PD-L1 to facilitate STT3 formation in the ER that induces the glycosylation of PD-L1 and promotes its stabilization. The combination of anti-IL-6 and anti-TIM3 antibodies has been demonstrated to improve the anti-tumor efficacy of CD8^+^ T cells, compared with anti-TIM3 monotherapy [153].

The ubiquitin-mediated proteasome degradation pathway extensively regulates PD-L1 expression, suggesting that there is a prospect of combining PD-L1 polyubiquitination regulators with ICIs as a potential therapy. E3 ligase β-TrCP catalyzes PD-L1 ubiquitination that degrades nonglycosylated PD-L1. Induction of β-TrCP expression by resveratrol reduces PD-L1 expression in vitro [152]. Speckle-type POZ protein (SPOP) is an E3 ubiquitin ligase that promotes the polyubiquitination and downregulation of PD-L1 through cyclin D-cyclin-dependent kinase 4 (CDK4). In a syngeneic mouse model of colon cancer, the CDK4/6 inhibitor palbociclib increases PD-L1 expression and synergizes with anti-PD-1 therapy to enhance therapeutic efficacy [162]. CKLF-like MARVEL transmembrane domain-containing 6 (CMTM6) prevents PD-L1 from undergoing polyubiquitination by E3 ubiquitin ligase STIP1 homology and U-box containing protein 1 (STUB1, [163, 164]. Conversely, COP9 signalosome complex subunit 5 (CSN5) is a deubiquitinase that inhibits the degradation of PD-L1, thereby enhancing PD-L1/PD-1 interactions that promote immune escape. Inhibition of CSN5 by curcumin reduces chronic inflammation-mediated PD-L1-based immunosuppression and synergizes with anti-CTLA-4 therapy in syngeneic mouse models of TNBC, melanoma, and colon cancer [165]. Ubiquitin-specific protease 22 (USP22) is a deubiquitinase that directly interacts with the C terminus of PD-L1. Genetic depletion of USP22 inhibits liver cancer growth, increases tumor immunogenicity, and improves the therapeutic efficacy of anti-PD-L1 therapy [166].

Palmitoylation is a recently discovered PTM of lipids that blocks PD-L1 ubiquitination and increases its stability. It has been found that zinc-finger DHHC-type-containing 3 (ZDHHC3) catalyzes the linking of palmitate to C272 on PD-L1. Inhibition of palmitoyltransferase by 2-bromopalmitate (2-BP) sensitizes tumor cells to destruction by T cells and inhibition of tumor growth [167, 168]. In summary, PTMs of PD-L1 that are targeted by drugs have displayed promise in preclinical trials. Whether they can represent candidates in combination therapies with anti-PD-(L)1 clinically still requires investigation.

### Precision medicine

Traditional drug-centered clinical trials for treatments of PC tend to identify patients with the same type of histology and provide specific drug regimens, while paying limited attention to the baseline characteristics, genomic subgroups, and tumor microenvironments of patients. Additional improvements in therapy require patient-centered trials in which patients with specific genetic and epigenetic information, RNA profiling, proteomic data, and immune markers are identified, allowing precision combination therapies [169, 170]. The molecular profiling of tissue and blood should be performed and evaluated both at the time of diagnosis and during treatment, to identify variable genetic and epigenetic diversity and monitor the response and resistance. Improved bioinformatics analysis is required to recognize key drivers of carcinogenesis. In PC, the interaction between tumor and stroma is also a significant factor influencing the efficacy of therapies, which require individualized analysis to identify molecular identity [171, 172]. Regarding precision therapy, optimization may require the use of treatment combinations that destroy PC using different mechanisms. Agents targeting KRAS, TP53, CDKN2A, and SMAD4, the four common mutations in PC, are still under development or under clinical investigation [172]. Considering the different modes of action of agents, their optimal dose, schedule, and sequence of administration will have to be determined scientifically and meticulously. Drug delivery, drug metabolism, and AEs for specific patients are also individual factors about which precision therapy will be considered [62].

## Conclusions

In the complex PC TME, combining anti-PD-(L)1 with chemotherapy, radiotherapy, molecularly targeted therapies or immunotherapies is challenged by the different characteristics of the different mechanisms and constraints in the TME. The mouse models, study sizes, and drug administration schedules varied across preclinical studies do not faithfully replicate the real world. Different patient selection, treatment dosing, and study designs have been used in clinical studies, resulting in distinctions in efficacy and AEs, which still require adjustment and optimization. For new combination strategies, drug repurposing proposes a promising direction with fewer development costs, shorter development timelines, and lower safety risks. Targeting post-translational modifications of PD-(L)1 has been investigated in preclinical trials and may be combined with anti-PD-(L)1 to improve future treatments. Moreover, precision medicine based on individualized genetic and epigenetic information, RNA profiling, proteomic data, and immune markers can help identify patients who will benefit from anti-PD-(L)1 and provide individually targeted combination strategies.

## Data Availability

Not applicable.

## A**bbreviations**

ADO: Adenosine
AE: Adverse effect
AMP: Adenosine monophosphate
AMPK: Adenosine-5′-monophosphate-activated protein kinase
APC: Antigen-presenting cell
ATM: Ataxia-telangiectasia mutated
BTK: Bruton’s tyrosine kinase
B3GNT3: β-1,3-N-acetylglucosaminyl transferase
CAF: Carcinoma-associated fibroblasts
CAR: Chimeric antigen receptor
CDK4: Cyclin D-cyclin-dependent kinase 4
CMTM6: CKLF-like MARVEL transmembrane domain-containing 6
CR: Complete response
CSF1: Colony-stimulating factor 1
CSN5: Complex subunit 5
CTLA-4: Cytotoxic T-lymphocyte antigen-4
CXCL12: CXC-chemokine ligand 12
CXCR4: CXC-chemokine receptor 4
DAMP: Damage-associated molecular pattern
DC: Dendritic cell
DCR: Disease control rate
DDR: DNA damage repair
DLT: Dose-limiting toxicity
dMMR: Mismatch repair-deficient
EGF: Epidermal growth factor
EMT: Epithelial-to-mesenchymal transition
ER: Endoplasmic reticulum
ERAD: ER-associated degradation
FAK: Focal adhesion kinase
FAP: Fibroblast activation protein
FDA: Food and drug administration
FKBP51: FK506 binding protein 51
FOLFIRINOX: Fluorouracil + leucovorin + irinotecan + oxaliplatin
GM-CSF: Granulocyte-macrophage colony-stimulating factor
GSK3β: Glycogen synthase kinase 3β
HA: Hyaluronic acid
HAS3: Hyaluronan synthase-3
HMGB1: High-mobility group protein B1
hNIS: Human sodium iodide symporter
ICI: Immune checkpoint inhibitor
IDO1: Indoleamine 2,3-dioxygenase 1
IFN: Interferon
IL-6: Interleukin-6
irAE: Immune-related adverse event
IRE: Irreversible electroporation
MDSC: Myeloid-derived suppressor cell
MHC: Major histocompatibility complex
MoDC: Monocyte-derived dendritic cell
ORR: Objective response rate
OS: Overall survival
OV: Oncolytic virus
PARP: Poly ADP-ribose polymerase
PC: Pancreatic cancer
PD: Progressive disease
PD-1: Programmed cell death protein 1
PD-L1: Programmed cell death ligand 1
PEGPH20: PEGylated recombinant hyaluronidase PH20
PFS: Progression-free survival
PR: Partial response
PSC: Pancreatic stellate cell
PTM: Post-translational modification
RT: Radiotherapy
SD: Stable disease
Sigma1: Sigma nonopioid intracellular receptor 1
siRNA: Small interfering RNA
SPOP: Speckle-type POZ protein
STT3: Staurosporine temperature sensitivity 3
STUB1: STIP1 homology and U-box containing protein 1
TAM: Tumor-associated macrophage
TCR: T cell receptor
TEAE: Treatment-emergent adverse event
Teff: Effector T cell
TGF-β: Transforming growth factor-β
TIM3: T cell immunoglobulin mucin-3
TME: Tumor microenvironment
TNBC: Triple-negative breast cancer
TNF: Tumor necrosis factor
Treg: Regulatory T cell
USP22: Ubiquitin-specific protease 22
VDR: Vitamin D receptor
ZDHHC3: Zinc-finger DHHC-type-containing 3
β-TrCP: β-transducin repeat-containing protein
2-BP: 2-bromopalmitate
